# A next-generation GMMA-based vaccine candidate to fight shigellosis

**DOI:** 10.1038/s41541-023-00725-8

**Published:** 2023-09-05

**Authors:** Omar Rossi, Francesco Citiulo, Carlo Giannelli, Emilia Cappelletti, Gianmarco Gasperini, Francesca Mancini, Alessandra Acquaviva, Maria Michelina Raso, Luigi Sollai, Renzo Alfini, Maria Grazia Aruta, Claudia Giorgina Vitali, Mariagrazia Pizza, Francesca Necchi, Rino Rappuoli, Laura B. Martin, Francesco Berlanda Scorza, Anna Maria Colucci, Francesca Micoli

**Affiliations:** 1 GSK Global Health Vaccines R&D (GVGH), Siena, Italy; 2 GSK Vaccines Srl, Siena, Italy; 3 Present Address: GSK Vaccines Srl, Siena, Italy; 4 Present Address: Imperial College, London, United Kingdom; 5 Present Address: Fondazione Biotecnopolo, Siena, Italy; 6 Present Address: US Pharmacopoeia, Rockville, Maryland USA

**Keywords:** Vaccines, Bacterial infection

## Abstract

Shigellosis is a leading cause of diarrheal disease in low-middle-income countries (LMICs). Effective vaccines will help to reduce the disease burden, exacerbated by increasing antibiotic resistance, in the most susceptible population represented by young children. A challenge for a broadly protective vaccine against shigellosis is to cover the most epidemiologically relevant serotypes among >50 *Shigella* serotypes circulating worldwide. The GMMA platform has been proposed as an innovative delivery system for *Shigella* O-antigens, and we have developed a 4-component vaccine against *S. sonnei*, *S. flexneri* 1b, 2a and 3a identified among the most prevalent *Shigella* serotypes in LMICs. Driven by the immunogenicity results obtained in clinic with a first-generation mono-component vaccine, a new *S. sonnei* GMMA construct was generated and combined with three *S. flexneri* GMMA in a 4-component Alhydrogel formulation (altSonflex1-2-3). This formulation was highly immunogenic, with no evidence of negative antigenic interference in mice and rabbits. The vaccine induced bactericidal antibodies also against heterologous *Shigella* strains carrying O-antigens different from those included in the vaccine. The Monocyte Activation Test used to evaluate the potential reactogenicity of the vaccine formulation revealed no differences compared to the *S. sonnei* mono-component vaccine, shown to be safe in several clinical trials in adults. A GLP toxicology study in rabbits confirmed that the vaccine was well tolerated. The preclinical study results support the clinical evaluation of altSonflex1-2-3 in healthy populations, and a phase 1–2 clinical trial is currently ongoing.

## Introduction

Shigellosis is the leading bacterial cause of diarrheal deaths worldwide, with 212,438 deaths, of which 30% are among children under 5 years of age and mainly in low- and middle-income countries (LMICs)^1^. In addition to morbidity and mortality due to diarrhea, repeated *Shigella* infections are a cause of long-term consequences, including stunting and cognitive impairment in children^2,3^. In high-income countries, populations at risk include soldiers, men who have sex with men, and travelers to LMICs^4^. Multidrug-resistant outbreaks have recently been reported in Seattle, WA, and in England^5,6^. Transmission of *Shigella* disease is facilitated by the low inoculum required to cause shigellosis (10–100 organisms)^7^, explaining the frequent failure of preventive sanitary and hygiene measures even in high-income countries^8^. Furthermore, antibiotic resistance in *Shigella* is increasing^9,10^, and the WHO’s Global Antimicrobial Resistance Surveillance System has listed *Shigella* as a priority pathogen for the development of new interventions^11^.

Currently, there are no licensed vaccines widely available against *Shigella*, but several candidates are under development^12^. The major challenge for the development of an effective vaccine against *Shigella* is the need to cover the most epidemiologically relevant serotypes. There are four different *Shigella* species—*S. dysenteriae*, *S. flexneri*, *S. boydii*, *S. sonnei*—divided into more than 50 different serotypes based on the outer polysaccharide antigen (O-antigen, OAg) of the lipopolysaccharide (LPS) displayed on the bacteria surface^13^. *S. flexneri* and *S. sonnei* account for nearly 90% of cases of shigellosis worldwide^14–16^ with a predominance of *S. flexneri* in LMICs^16,17^ and *S. sonnei* in high-income countries^7^. Immunity to *Shigella* appears to be serotype-specific, thus indicating that the OAg is the protective antigen, and many *Shigella* vaccine candidates are OAg-based^18^.

*S. flexneri* serotypes, other than *S. flexneri* 6, share a common backbone consisting of repeats made of three rhamnose residues and one N-acetylglucosamine^19,20^. The diversity of *S. flexneri* serotypes is due to the modification of this common OAg backbone with glucosyl and/or O-acetyl groups as a result of bacteriophage infection and acquisition of OAg-modifying enzymes^19,21^. alternatively, the OAg repeat of *S. sonnei* is a disaccharide of FucNAc, 2-acetamido-4-amino-2,4,6-trideoxy-D-galactose and AltNAcA, 2-amino-2-deoxy-L-altruronic acid. There are two ways of achieving broad coverage against *Shigella*: (1) by incorporating multiple serotypes in the vaccine; (2) by choosing serotypes that induce cross-reacting antibodies. If the cross-reaction observed in animals can be extrapolated to humans, multi-component vaccines against *S. sonnei* and the most commonly circulating *S. flexneri* serotypes are expected to achieve approximately 65% coverage, which could further increase to over 85% depending upon the degree of cross-protection elicited against *S. flexneri* strains not contained in the vaccine^16,22,23^.

We have used GMMA as an innovative delivery system for *Shigella* OAg. Gram-negative bacteria (e.g., *Shigella*) naturally shed outer membrane exosomes into their surrounding environment. GMMA are outer membrane exosomes released from genetically engineered bacteria. The genetic modifications include the disruption of the linkages between the outer membrane and the peptidoglycan or inner membrane (e.g., *tolR* knock-out [KO])^24^ to greatly increase GMMA release. Modification of the Lipid A structure, e.g., by deletion of *msbB* or *htrB* genes^25–27^, results in reduced stimulation of innate immune response by GMMA. Based on preclinical results in mice, Alhydrogel does not increase the functional IgG response induced by GMMA^28,29^, but GMMA were tested in clinical trial adsorbed on aluminum hydroxide to potentially enhance their tolerability^30^. Purification of GMMA from high-density fermentation cultures of the genetically modified bacteria is efficient, simple and rapid^31,32^. A large body of preclinical data have been collected showing that GMMA are highly immunogenic and able to elicit protective responses in animals^33^.

Ease of manufacture and high immunogenicity make GMMA a technology platform suited for vaccines designated to LMICs. Human proof of concept with the GMMA platform was obtained with a first-generation *S. sonnei* GMMA vaccine candidate, 1790GAHB. In phase1 and phase2 studies in healthy European and African adults, 1790GAHB was well tolerated at doses up to 6 µg OAg/100 µg protein adsorbed to Alhydrogel. Intramuscular administration induced a substantial increase in specific antibodies compared to pre-vaccination levels and good boosting of both vaccine and natural immunity^30,34,35^. Antibodies generated were able to kill bacteria in vitro in the presence of complement^36,37^. However, a phase 2b human challenge trial conducted with a 1.5 µg OAg/25 µg protein dose demonstrated that a higher OAg dose is likely required to achieve clinical protection^38^. Hence, here we report the generation of a new *S. sonnei* construct (2929-GMMA) that harbors a 10-fold higher OAg content (relative to protein and lipid A content) compared to the original *S. sonnei* GMMA (1790-GMMA). The new construct has been incorporated in a 4-component formulation, called altSonflex1-2-3, together with GMMA from 3*S. flexneri* strains, 1b, 2a and 3a, selected to be epidemiologically relevant^15^ and for their ability to induce cross-reactive antibodies with the potential to elicit broad protection against the most prevalent *Shigella* serotypes^39^. The preclinical data package collected and presented here has allowed us to start a phase1/2 trial (clinicaltrial.gov NCT05073003) that is currently ongoing.

## Results

### Generation of an alternative *S. sonnei* GMMA

**Fig. 1:**
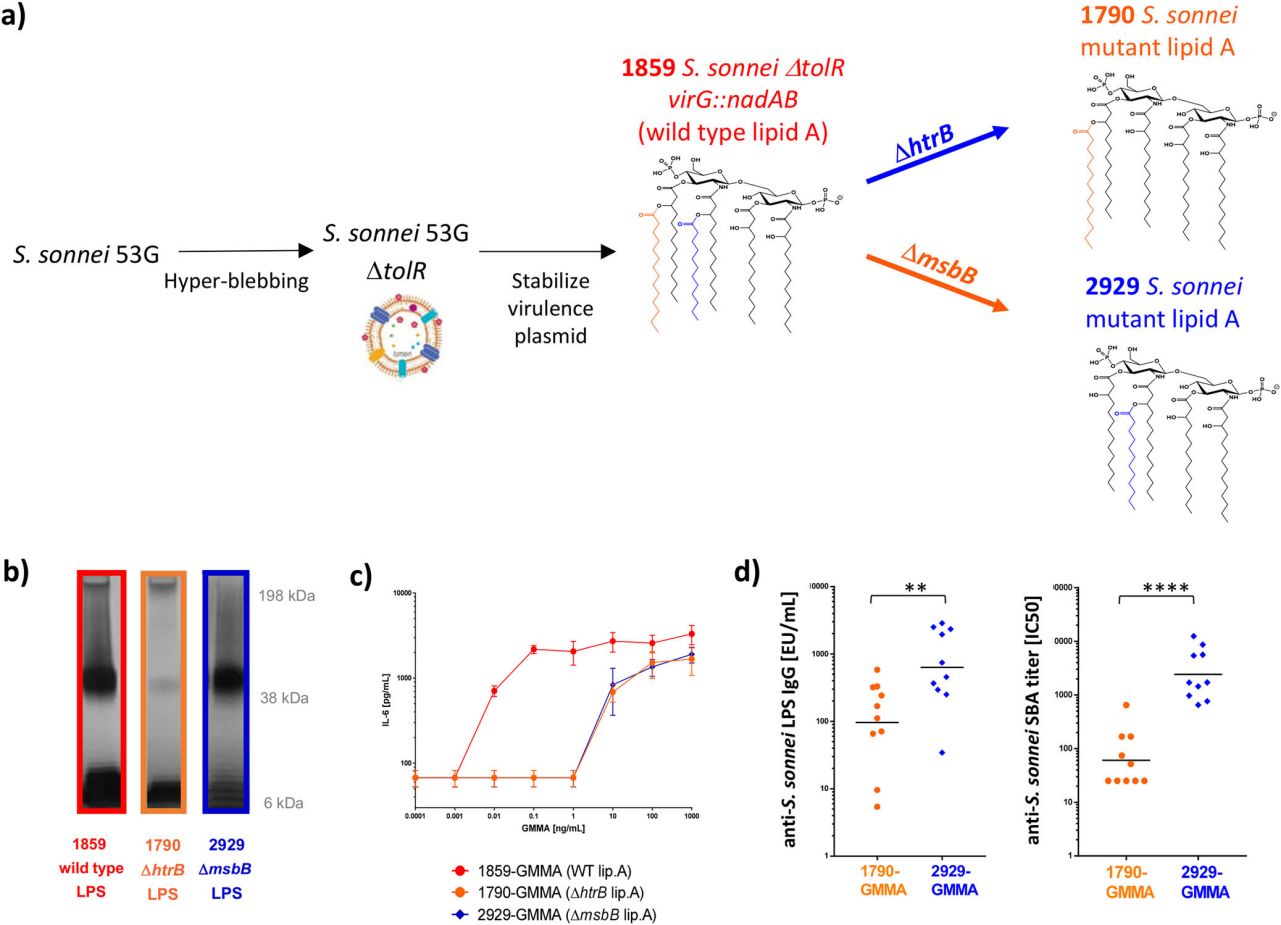
Generation of *S. sonnei* 1790-GMMA and 2929-GMMA and their comparison. **a** Strategy used for the production of GMMA with mutated lipid A; **b** SDS-PAGE silver stained of LPS extracted from GMMA producing bacteria normalized at same OD; Gels derive from the same experiments and have been processed in parallel. **c** IL-6 release by human PBMC upon stimulation with GMMA with different lipid A structures (samples were compared at same GMMA protein concentration); **d** anti-LPS IgG response and SBA titers elicited in mice by 2929-GMMA vs 1790-GMMA when compared at same total protein dose of 0.01 μg. Dots correspond to responses of single mice and bars to geometric means. CD1 mice were immunized IM at days 0 and 28, and sera were analyzed at day 42. Geometric mean (bar) is reported with individual values (dots). Statistically significant differences by Mann–Whitney two-tailed test are indicated (** if *p* < 0.01, **** if *p* < 0.0001).

1790-GMMA formulated in the first-generation *S. sonnei* mono-component vaccine candidate, 1790GAHB, had an OAg to protein weight ratio of 0.03 (Supplementary Table 1). We had previously observed a strong decrease in OAg amount on GMMA after lipid A de-acylation in the GMMA producer strain. Indeed, *S. sonnei* 53G wild-type strain, with stabilized virulence plasmid encoding the OAg operon, after deletion of *tolR* resulted in hexa-acylated lipid A GMMA (1859-GMMA) characterized by an OAg to protein weight ratio of 0.21 (Supplementary Table 1). However, after *htrB* deletion, introduced to mutate the wild-type hexa-acylated in a less reactogenic penta-acylated lipid A, the OAg to protein ratio was reduced (Supplementary Table 1)^31^. With the aim to generate a *S. sonnei* GMMA with higher OAg density compared to the first generation 1790-GMMA, the hyper-blebbing strain was deleted of the chromosomal gene *msbB1* and of the plasmid-borne *msbB2* gene (Fig. 1a). Both *htrB* and *msbB genes* were previously reported to contribute to the full hexa-acylated lipid A biogenesis in *E. coli*^40^. The *msbB* mutated strain (2929-GMMA) retained an OAg density similar to the GMMA with wild-type lipid A, 10-fold higher compared to 1790-GMMA. This was first shown by SDS-PAGE silver staining analysis of LPS extracted by the different mutants (wild type*, ΔhtrB, ΔmsbB* in Supplementary Table 1; Fig. 1b and Supplementary Fig. 1 when normalized to total protein content). A more detailed analysis of the OAg populations was performed after their acetic acid extraction and isolation from GMMA^41^. All three GMMA displayed a capsular polysaccharide (Group 4 Capsule, G4C^42^) overlapping with high molecular weight (HMW) OAg, and a medium molecular weight (MMW) OAg having similar size but different relative abundance^43^. In particular, 1859-GMMA and 1790-GMMA showed a prevalence of the MMW OAg population around 20–50 kDa (~75% in weight on total sugar), while 2929-GMMA expressed similar levels of G4C+HMW OAg at 200–240 kDa and MMW OAg (48 and 33% respectively). All GMMA also contained an OAg population at low molecular weight (LMW), composed of core only and core plus few OAg repeats (Supplementary Table 1). Dynamic light scattering (dls) analysis resulted in a bigger average size of 2929-GMMA particles (Z average diameter of 160.4 nm) compared to 1859-GMMA (103.4 nm) and 1790-GMMA (118.9 nm), very likely reflecting the higher relative amount of G4C/HMW OAg vs MMW OAg in 2929-GMMA^41^.

Lipid A to protein ratio, expressed in nmol lipid A per mg of protein, was higher for wild-type lipid A 1859-GMMA (287 nmol/mg) than for 1790-GMMA (197 nmol/mg) and 2929-GMMA (144 nmol/mg), that showed similar ratios (Supplementary Table 1). 2929-GMMA contain a different type of penta-acylated lipid A structure with respect to 1790-GMMA: compared to the wild-type lipid A, 2929-GMMA lipid A does not contain the myristic fatty acid chain, while the 1790-GMMA lipid A does not contain the lauric fatty acid chain (Fig. 1a). Despite this difference, when these GMMA were compared in a Monocyte Activation Test (MAT) at same protein dose, 1790-GMMA and 2929-GMMA stimulated similar levels of IL-6 release from human peripheral blood mononuclear cells (PBMC), which was several hundred-fold lower than the corresponding GMMA with wild-type lipid A (1859-GMMA; Fig. 1c).

As expected, when immunizing mice at the same protein dose of 10 ng, new-generation 2929-GMMA elicited anti-*S. sonnei* LPS IgG and SBA titers significantly higher than first-generation 1790-GMMA, in line with 10-fold higher OAg amount despite similar lipid A content (Fig. 1d).

### Generation of *S. flexneri* 1b, 2a and 3a GMMA

Genetic manipulations used for *S. sonnei* GMMA were similarly applied to *S. flexneri* 1b, 2a and 3a wild-type strains for corresponding GMMA generation. The LPS of *S. flexneri* GMMA with modified lipid A contain the same penta-acylated population of *S. sonnei* 2929-GMMA (lacking the myristic fatty acid chain compared to wild type) but also an additional population of hexa-acylated non-wild-type form of lipid A due to palmitoylation of the penta-acylated species by the action of the palmitoyl transferase enzyme PagP (Supplementary Fig. 2). Despite this difference, all genetically modified lipid A structures of *S. flexneri* GMMA possess the reduced ability to induce proinflammatory cytokine release from human PBMC in vitro by MAT when compared with GMMA containing wild-type lipid A and at a level similar to 1790-GMMA, which has been tested in clinical trials in adult populations without safety concerns^30,31^ (Supplementary Fig. 3).

Also, the process for GMMA production and purification was easily extended from *S. sonnei* to *S. flexneri* constructs. All *S. flexneri* GMMA had a homogeneous and similar particle size, with Z-average diameter ranging from 80 to 110 nm and polydispersity index (PDI) of 0.14–0.18. The OAg to protein weight ratio was close to 1 for all *S. flexneri* GMMA and higher than for *S. sonnei* 2929-GMMA (Supplementary Fig. 4). Lipid A to protein ratio was higher for *S. flexneri* 1b and 3a (~0.4 nmol/µg) compared to *S. flexneri* 2a (0.25 nmol/µg) and *S. sonnei* 2929-GMMA (0.14 nmol/µg) (Supplementary Table 2).

All GMMA showed the presence of a MMW OAg close to 14 kDa and of a LMW OAg close to 2 kDa characterized by the presence of very few repeating units attached to the core. Only *S. flexneri* 2a GMMA showed the presence of an OAg population at a higher molecular weight of approximately 47 KDa, which was 56% in weight of the amount of HMW+MMW OAg (Supplementary Table 2, Supplementary Fig. 4). Supplementary Fig. 4 also reports structures of *S. sonnei* and *S. flexneri* 1b, 2a and 3a OAg. *S. flexneri* OAg differs in the position of glucose and/or O-acetyl groups on the same backbone. NMR analysis (Supplementary Fig. 5) revealed that *S. flexneri* 1b OAg had an average of 1.8 O-acetyl groups per repeating unit on RhaI (2-OAc) and RhaIII (3/4-OAc), *S. flexneri* 2a OAg had an average of 1.4 O-acetyl groups per repeating unit on GlcNAc (6-OAc) and RhaIII (3/4-OAc) and *S. flexneri* 3a OAg had an average of 1 O-acetyl group per repeating unit on RhaI (2-OAc). Different GMMA batches have been generated at 30 L scale to confirm the robustness and reproducibility of the process (Supplementary Tables 3 and 4).

### 4-component altSonflex1-2-3 formulation

*S. sonnei* (2929-GMMA), *S. flexneri* 1b, 2a and 3a GMMA were normalized based on OAg content and were adsorbed on Alhydrogel. Importantly, the highest envisaged human dose, corresponding to 15 μg of each OAg, has total protein and lipid A content close to 100 μg and 30 nmol, respectively, lower than the corresponding contents of the highest first-generation 1790GAHB dose shown to be well-tolerated in adults (175 μg protein by Lowry and 47 nmol lipid A)^30^. An analytical panel was developed to characterize the 4-component formulation. High-performance anion-exchange chromatography/pulsed amperometric detection (HPAEC-PAD) allows the determination of *S. sonnei* OAg and *S. flexneri* total OAg quantification. All *S. flexneri* OAg are constituted by the same sugar monomers, and discrimination of individual components is not possible through this analysis. For this reason, the Formulated Alhydrogel competitive-ELISA (FAcE) assay, based on the recognition of each OAg on Alhydrogel by specific corresponding monoclonal antibodies, has been used^44^. This assay allows the determination of each GMMA in the 4-component adsorbed formulation. D[4,3] and D(90) by light scattering (indicating respectively the weighted mean size by volume and the size value below which 90% of the population is included) are used to determine Alhydrogel formulation size. Micro BCA and HPLC-RP/MS analyses are used to verify the presence of GMMA not adsorbed on Alhydrogel by determining total protein content and lipid A content, respectively. These were confirmed to be at low levels in all preparations (≤10% total protein and total lipid A). Supplementary Table 5 reports the characterization of multiple altSonflex1-2-3 batches, confirming the robustness of the process and comparability of results. Stability data in real time (2–8 °C up to 24 months) and accelerated (25 and 40 °C for up to 56 days) conditions collected so far support a shelf-life assignment of 36 months to altSonflex1-2-3.

### Immunogenicity of next-generation 4-component GMMA-based vaccine candidate altSonflex1-2-3

**Fig. 2:**
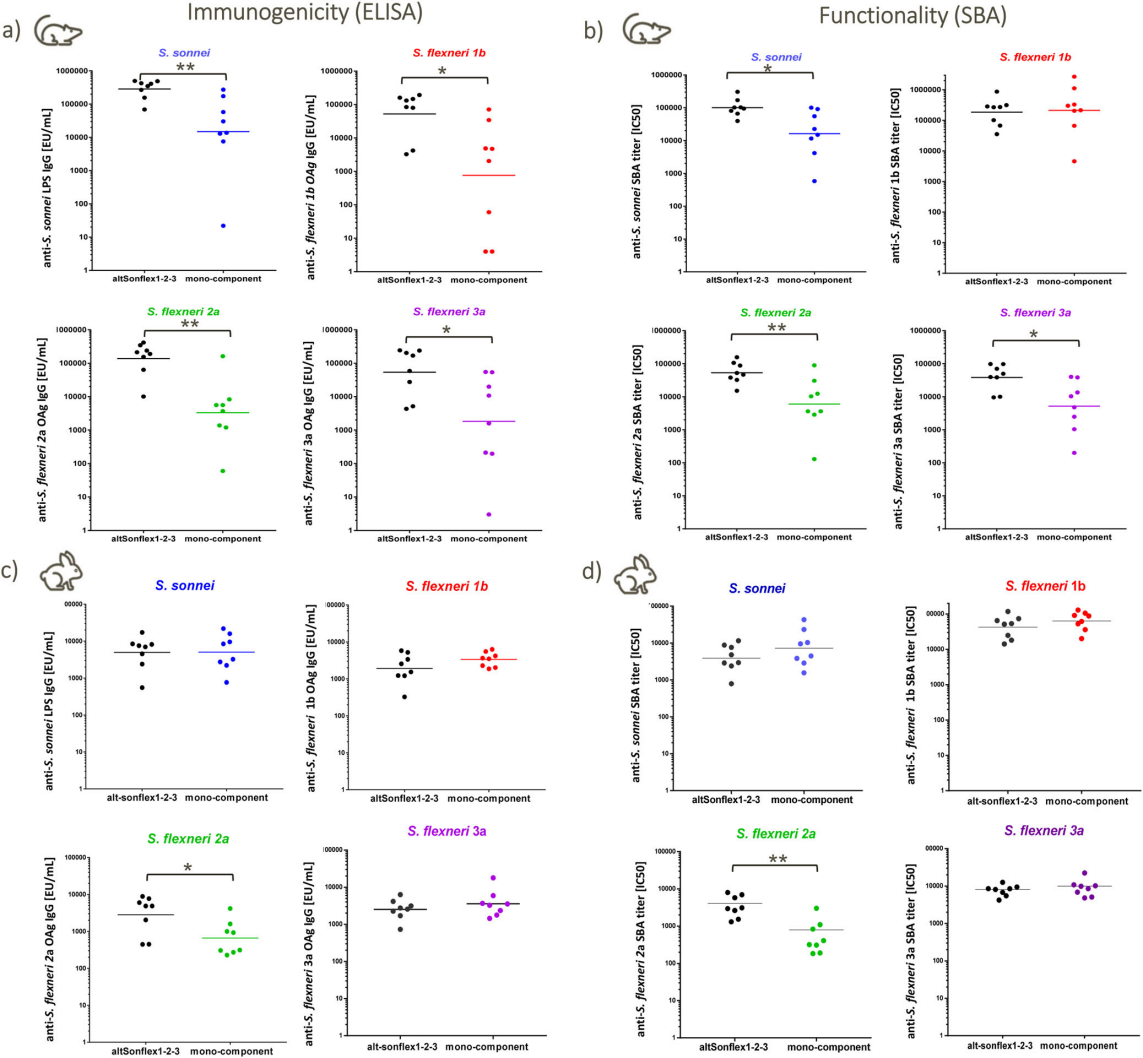
Serotype-specific OAg IgG and SBA titers against each serotype in the vaccine induced by altSonflex1-2-3 in mice or rabbits compared to corresponding mono-component formulations. **a** Anti-OAg IgG in mice; **b** SBA titers in mice; **c** anti-OAg IgG in rabbits; **d** SBA titers in rabbits. CD1 mice were immunized IP with 9 ng each OAg per dose; rabbits were immunized IM with 1.5 μg each OAg per dose. Immunizations were performed on days 0 and 28, and sera were analyzed on day 42. Geometric mean (bar) is reported for all groups together with individual values (dots). Statistically significant differences by Mann–Whitney two-tailed test are indicated (* if *p* < 0.05, ** if *p* < 0.01).

To verify that the combination of four different GMMA did not negatively affect the immune response elicited by each single component, altSonflex1-2-3 was tested in mice in comparison to the corresponding mono-component formulations. No negative antigen interference was observed across serotypes, both in terms of IgG response at 27 days post first injection and 14 days post second injection and serum bactericidal activity against the serotypes included in the vaccine 14 days post second injection (Fig. 2a, b, Supplementary Fig. 6). In most cases, the functional IgG response induced by altSonflex1-2-3 vaccine was higher than the individual GMMA formulations. GMMA elicited a significant anti-OAg IgG response also at the very low OAg dose tested of 9.4 ng. Immunogenicity results were also confirmed in rabbits, where each GMMA was tested at a 1.5 μg OAg dose. Two weeks after the second injection given on day 28, all GMMA elicited similar anti-OAg functional IgG whether tested as a single component formulation or in the 4-component formulation, with the exception of *S. flexneri* 2a GMMA that elicited higher IgG response and SBA titers in altSonflex1-2-3 formulation compared to *S. flexneri* 2a GMMA formulation alone (Fig. 2c, d).

When tested in mice at different doses (from 0.58 to 37 ng of each OAg with a 4-fold dilution step), altSonflex1-2-3 vaccine candidate induced a significant anti-OAg IgG specific response against all four serotypes already after one injection and boosted the response 14 days after re-vaccination with a 1-month interval. The ability of the vaccine candidate to elicit anti-OAg IgG response in a dose-dependent manner was verified after both the first and second injections (Supplementary Fig. 7). Functional antibodies against the serotypes present in the vaccine were elicited in a dose-dependent manner (Supplementary Fig. 8).

**Fig. 3:**
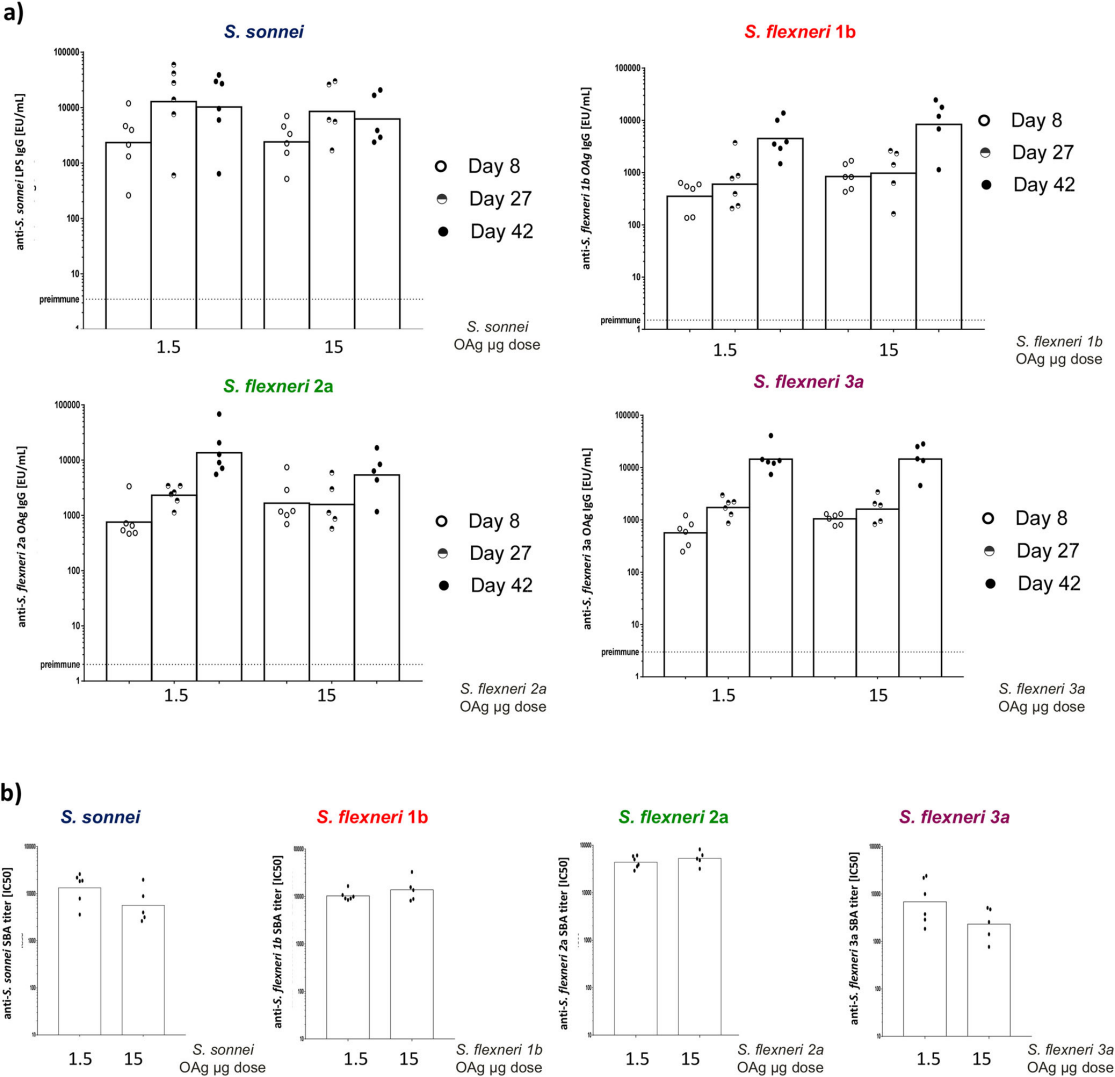
Serotype-specific OAg IgG and SBA titers against each serotype in the vaccine induced byaltSonflex1-2-3 in rabbits at full human dose and 1/10 of the full human dose. Anti-OAg IgG responses (**a**) and bactericidal titers against the serotypes included in the vaccine candidate (**b**) elicited in rabbits by altSonflex1-2-3 at full human dose of 15 μg each OAg and at 1/10 of such dose. New Zealand rabbits were immunized IM at days 0 and 28, and sera were analyzed at days 8 (ELISA), 27 (ELISA) and 42 (ELISA and SBA). Geometric mean (bar) is reported for all groups together with individual values (dots).

**Fig. 4:**
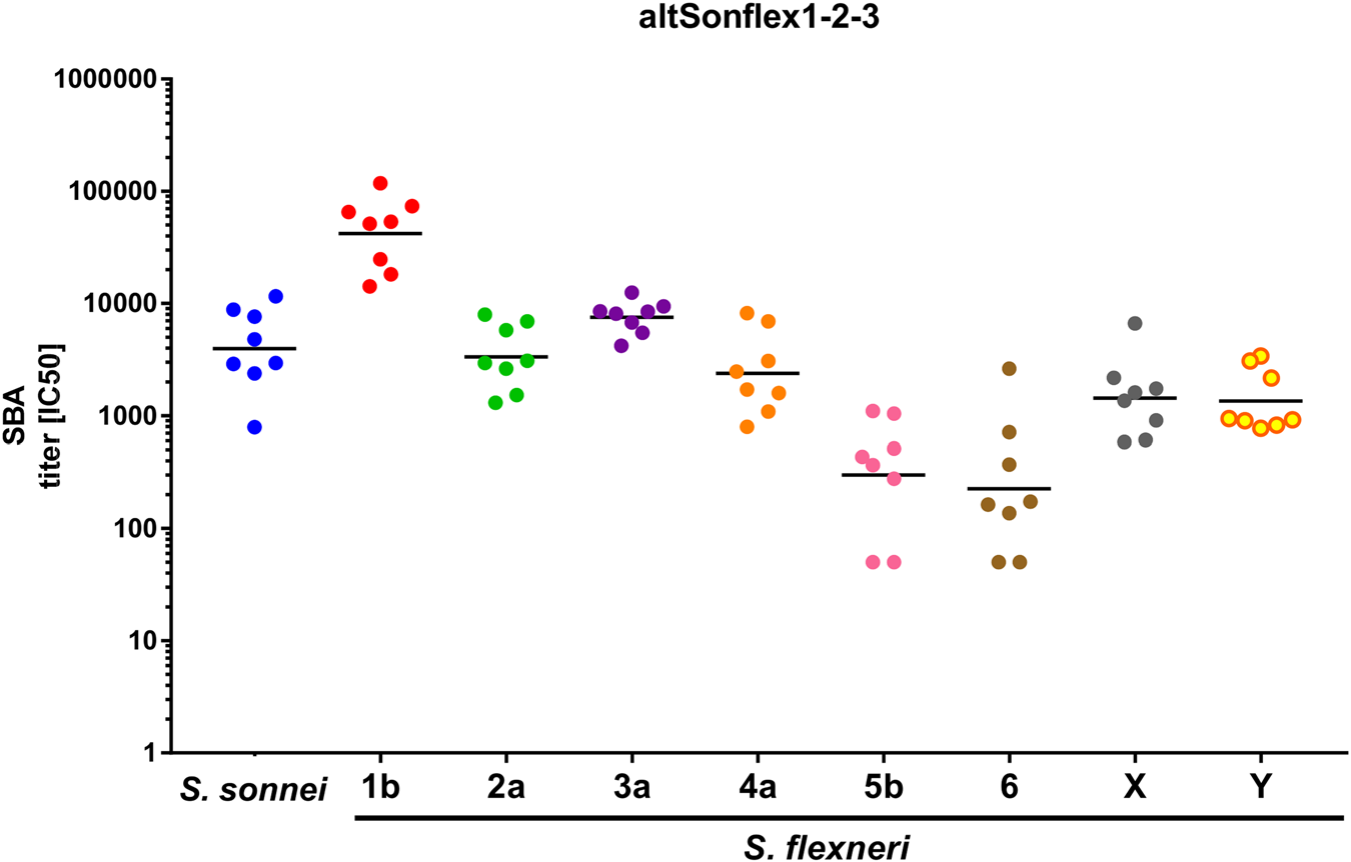
SBA activity against epidemiologically relevant *Shigella* wild-type strains. Sera at day 42 from rabbits immunized IM at days 0 and 28 with 1.5 μg each OAg were tested. Geometric mean (bar) is reported for all groups together with individual values (dots). A titer of 50 was assigned to nonresponders.

altSonflex1-2-3 was also tested in rabbits at the highest human dose envisaged of 60 μg total OAg (15 μg each). All GMMA components elicited significant anti-OAg-specific IgG response already at 8 days after the first injection, which increased further 14 days after the second injection given on day 28 for all *S. flexneri* GMMA, but not for *S. sonnei* GMMA (Fig. 3a). Similar responses were obtained at all time points with 1/10 of the dose, both in terms of ELISA (Fig. 3a) and SBA titers at day 42 (Fig. 3b). Antibodies elicited by altSonflex1-2-3 were also tested for their ability to bind other *S. flexneri* O-antigens epidemiologically relevant not present in the vaccine (namely *S. flexneri* serotypes 4a, 5b, 6, X, Y). Both mouse and rabbit sera showed binding to the panel of heterologous *S. flexneri* O-antigens/wild-type strains tested (Supplementary Fig. 9): single sera tested were all positive compared to pre-immune sera. In mice, sera showed stronger binding for the serotypes included in the vaccine, while sera from rabbits showed more homogeneous binding. Interestingly, rabbit sera were also able to kill such strains in SBA (Fig. 4).

### Safety profile of altSonflex1-2-3

Immunogenicity studies performed in mice and rabbits showed that altSonflex1-2-3 was immunogenic in both animal species. These results supported a GLP toxicology study in rabbits: four intramuscular administrations of the full human dose of altSonflex1-2-3 at 2-week intervals to New Zealand White rabbits were well tolerated.

Findings were of low severity, generally reversible and represented a physiological inflammatory reaction following the immune stimulation in response to the vaccine. Also, IL-6 release from human PBMC by MAT was similar after stimulation with altSonflex1-2-3 at the full human dose of 15 µg of each OAg and 1790GAHB clinical batch containing the maximum dose of 6 µg OAg, when the dilutions starting from the full human doses were compared (Supplementary Fig. 10).

## Discussion

Several vaccine candidates currently in development against *Shigella* are OAg-based^18^. According to sero-epidemiological studies and challenge–rechallenge studies^45–47^, clinical infection with *Shigella* strains confers serotype-specific immunity. Also, live oral vaccines^48^ and OAg glycoconjugates^49^ that have conferred protection in randomized controlled field trials have confirmed the OAg as potential protective antigens for *Shigella*. However, a *S. sonnei* OAg conjugate was effective in Israeli adults but failed to elicit protection in younger children^50^. We have used GMMA as delivery system for *Shigella* OAg. Studies in mice with *S. flexneri* 6 and *Salmonella* Typhimurium and Enteritidis GMMA have shown the ability of GMMA to elicit higher functional IgG responses than traditional OAg glycoconjugates^28,29^. GMMA combine T-cell help with particulate size and the presence of PAMPs with the potential for a strong immune response in humans^51^. GMMA resemble the outermost layer of bacteria, presenting multiple OAg in their native environment and overcoming reactogenicity issues of whole cell vaccines^33^. In GMMA, the lipid A is mutated to reduce endotoxicity^25^. However, in the original *S. sonnei* 1790-GMMA construct, *htrB* deletion strongly impacted the OAg density on GMMA surface^31^. When tested in a Controlled Human Infection Model (CHIM) study at 1.5 µg OAg dose, the 1790GAHB formulation failed to confer protection against shigellosis^38^. Nevertheless, the study confirmed a critical role for anti-LPS IgG antibodies as anti-*S. sonnei* LPS serum IgG were higher among participants who did not develop shigellosis compared to cases following the challenge dose. Insufficient anti-LPS IgG response was considered as the main root cause for the lack of efficacy, suggesting the need for an enhanced *S. sonnei* OAg dose. The appropriateness of increasing the OAg dose, with respect to the 1.5 μg OAg tested in the challenge trial with 1790GAHB, is supported by previous clinical trials with the 1790GAHB vaccine candidate, showing dose response^35,36^, and by the higher OAg content used for other *Shigella* OAg candidate vaccines. The Flexyn2a vaccine, a bioconjugate produced in *E. coli* by LimmaTech Biologics and containing 10 µg of *S. flexneri* 2a OAg linked to *r*EPA showed a protective efficacy point estimate of 51.7% against severe diarrhea in a CHIM trial. The SF2a-TT15, containing 10 μg oligosaccharide conjugated to tetanus toxoid, was more immunogenic at the 10 µg dose compared to the 2 µg dose in healthy adults in Israel^52^. Also, the US NIH *S. sonnei* conjugate vaccine showing 74% protective efficacy against shigellosis in adults^53^ contained 25 µg of OAg.

Here a different lipid A mutation strategy was applied, through the deletion of genes encoding for late acyltransferase *msbB*, resulting in a GMMA with a different penta-acetylated lipid A structure compared to the 1790-GMMA, which did not result in a reduction of OAg density on the bacterial surface. In line with that, the new construct, 2929-GMMA, compared at the same protein and lipid A dose, with a similar safety profile in vitro, was more immunogenic than 1790-GMMA. Also, the higher OAg content on *S. sonnei* 2929-GMMA has allowed four different *Shigella* GMMA to be combined in altSonflex1-2-3 at 15 µg OAg dose each, maintaining total lipid A and protein content inferior to the maximum amount already safely tested with 1790GAHB in adult populations. This dose is 10-fold higher compared to the quantity tested in the CHIM study with 1790GAHB and of a similar OAg quantity compared to the conjugate vaccine candidates mentioned above.

GMMA is a technology platform characterized by simplicity of manufacturing and potential for low cost of goods^32^. Experience with first-generation *S. sonnei* 1790-GMMA allowed to rapidly produce *S. flexneri* GMMA: similar fermentation and purification conditions were adopted, resulting in high purity and yield of GMMA, corresponding to 30 mg OAg/L of bacterial culture for *S. sonnei* 2929-GMMA and to 250 mg OAg/L of bacterial culture for *S. flexneri* GMMA.

A complete analytical panel was developed to allow characterization of GMMA and Alhydrogel formulated GMMA and a series of studies have been performed to identify GMMA critical quality attributes^43,54^. This has allowed rapid definition of characteristics to include in the release panel of *Shigella* GMMA and altSonflex1-2-3 and to follow during stability studies.

The new generation 4-component formulation was immunogenic both in mice and rabbits. Strong antigen-specific IgG responses were observed shortly after the first immunization in both animal species. Following a second immunization, the level of specific OAg antibodies increased substantially in mice and in rabbits. Only for *S. sonnei* GMMA an absence of booster was observed in rabbits after an interval of 28 days between the two immunizations. This reflects the lack of an increase in the response observed with 1790GAHB in adults^55^. For glycoconjugates, a combination of different components can result in impairment of the immune response, as measured by lower antibody levels^56^. Importantly the combination of multiple GMMA resulted in no immunologic interference in either animal model tested^56^.

Antibodies elicited by altSonflex1-2-3 were bactericidal. In particular, in rabbits, even 1/10th of the full human dose was able to induce antibodies not only able to induce killing of the strains contained in the vaccine but also a broad panel of strains which already account for >80% of the most sero epidemiologically relevant *Shigella* strains. The human immune system can provide protection against *Shigella* via multiple mechanisms that are not yet fully elucidated. Antibodies to LPS can fix complement and kill target bacteria. Thus, a serum bactericidal assay that replicates this killing process in vitro would allow the detection of antibodies that could display bactericidal activity in vivo and contribute to protective immunity. Recent studies have proposed SBA as a critical mechanism by which anti-*Shigella* LPS IgG can protect against infection^57,58^.

Furthermore, bactericidal activities have been detected in sera from infected individuals living in regions of *Shigella* endemicity^59–62^. In previous studies, we^38,63^ have verified that anti-GMMA protein antibodies are not functional in SBA; therefore, the cross-functionality observed in this study against *S. flexneri* strains not included in the vaccine should be OAg mediated. How results in animals will translate in humans is difficult to predict, and we have not tested the ability of altSonflex1-2-3 to protect in challenge animal models^64^, which have been only recently successfully established, and anyway, not for all the serotypes included in our vaccine candidate. However, sera from clinical trials will be analyzed soon for cross-functionality against the panel of heterologous *S. flexneri* strains. In case epidemiologically relevant *S. flexneri* strains will not be covered by the current formulation, the platformability of the GMMA technology will allow to rapidly add novel components to the current formulation or modify the current composition.

altSonflex1-2-3 has also shown a good safety profile in both in vivo (toxicology study) and in vitro assays. The use of an in vitro test is preferred to the rabbit pyrogenicity test (RPT) in line with the ‘3Rs’ principle of Replacement, Reduction and Refinement^65^. Over the years, especially for products that are inherently pyrogenic as GMMA and containing wild type or mutated lipid A LPS molecules, the use of MAT has increased to overcome drawbacks of RPT, including the use of animals and associated high biological variability, as well as species-dependent differences in TLR specificity, which may affect the translatability to humans of the results obtained in rabbits^66,67^.

The preclinical data package described has allowed to proceed with altSonflex1-2-3 testing in a phase 1/2 clinical trial (clinicaltrial.gov NCT05073003): the vaccine has completed administration in European adults and has entered an age de-escalation trial in Kenya, a country where shigellosis is endemic. The results of this trial may allow the selection of the most appropriate dose for further vaccine development in infants 9 months of age, the target age group for a *Shigella* vaccine in LMICs.

## Methods

### *S. sonnei* strain generation

*S. sonnei* 53G was chosen as the parent strain. Mutants were generated by using a three-step PCR protocol to fuse the gene upstream and downstream regions to the resistance gene. The linear fragment was used to transform recombination-prone *Shigella* carrying pAJD434 to obtain the respective deletion mutant. *S. sonnei* strain NVGH1859 (*S. sonnei* 53G *ΔtolR::kan ΔvirG::nadAB*) was obtained first by replacing the *tolR* gene with the kanamycin resistance gene *kan*, as described by Berlanda Scorza et al.^24^. Subsequently, the virulence plasmid-encoded *virG* gene was replaced with the *nadA* and *nadB* genes from *E. coli*, as described by Gerke et al.^31^. *S. sonnei* strain NVGH2929 (*S. sonnei* 53G *ΔtolR::kan ΔvirG::nadAB ΔmsbB2::cat ΔmsbB::erm*) was generated from NVGH1859 by replacing the *msbB1* and *msbB2* genes with the erythromycin and chloramphenicol resistance genes *erm* and *cat*, as described by Berlanda Scorza et al.^24^ and Mancini et al.^63^.

### *S. flexneri* 2a and 3a strain generation

*S. flexneri* 2a 2457T and *Shigella flexneri* 3a 6885 were chosen as parent strains. Before the generation of mutants, white colonies for each strain were selected on Congo red agar, indicating the loss of the virulence plasmid pINV. The curing of pINV was confirmed by the absence of the origin of replication (ori) or plasmid-encoded genes using PCR. *S. flexneri* 2a strain NVGH2404 (*S. flexneri* 2457T *ΔtolR::kan, ΔmsbB::cat*) and *S. flexneri* 3a strain NVGH2766 (*S. flexneri* 6885 *ΔtolR::kan, ΔmsbB::cat*) were obtained as previously described. Briefly, the *tolR* gene was replaced with the kanamycin resistance gene *kan* using the same strategy and primers described for *S. sonnei* by Berlanda Scorza et al.^24^. Subsequently, the *msbB* gene was replaced with the chloramphenicol resistance gene *cat*, using the same strategy and primers described by Rossi et al.^25^ Contrarily to *S. sonnei* strain NVGH2929, the *msbB2* mutation was not needed as the gene is located on the cured virulence plasmid.

### *S. flexneri* 1b strain generation

*Shigella flexneri* 1b Stansfield was chosen as parent strains. As for *S. flexneri* 2a and 3a, a white colony was selected on Congo red agar before the start of the genetic modification, indicating the loss of the virulence plasmid pINV. *S. flexneri* 1b strain NVGH2858 (*S. flexneri* Stansfield *ΔtolR::frt ΔmsbB1a::frt ΔmsbB1b::frt*) was prepared by adapting the methods described in Datsenko et al.^68^. Briefly, the *tolR* gene and two chromosomal copies of the *msbB* genes were replaced with the kanamycin resistance gene *kan*, using the primers listed in Supplementary Table 6. Removal of the antibiotic selective marker was performed after each gene deletion using the plasmid pCP20.

### *S. sonnei* and *S. flexneri* 1b, 2a and 3a GMMA production and purification

For each production batch, the *Shigella* strains are grown in a shake flask from a Research or GMP cell bank in a defined medium (DM^31^) at 30 °C with agitation (200 rpm), starting from an appropriate optical density measured at 600 nm (OD600) until the culture reaches an OD600 equal to 4.5 ± 2 after 6–7 ± 3 h. At the end of the inoculum flask incubation, the inoculum culture is transferred into the bioreactor (30 L scale). The inoculum size is 2–4%. The fermentation conditions are controlled: pH 6.7 kept by addition of 25–28% NH_4_OH, 30 °C, dissolved oxygen kept at 30%, airflow 15 to 30 standard liters per minute (SLPM), stir speed 50–800 rpm (cascade mode) until the final OD600 of 35–40 is reached. *S. flexneri* 1b, 2a and 3a GMMA released into the fermentation broth are purified using two consecutive Tangential Flow Filtration (TFF) steps: the first one is microfiltration (0.2 μm cut-off) where the broth is concentrated three times then diafiltered five times against buffer (TMP 1.0 ± 0.1 barg) and in which the culture supernatant containing the GMMA is separated from the bacteria. The second one is ultrafiltration (300 KDa cut-off), where GMMA solution is concentrated 10 times, then diafiltered 20 times against saline solution (TMP 0.5/1.0 barg) and in which the GMMA are separated from soluble proteins^31^. *S. sonnei* process differs from the process described above for the following steps: there is a feeding of amino acids and glucose solution during the first 10 h of the fermentation and an addition of benzonase (100,000U) when fermentation reaches OD 14 ± 2. At the end of the fermentation step, the broth is harvested by centrifugation (12,220×*g* for 15 min). The supernatant post centrifugation containing the GMMA is filtered by using a 0.8/0.65-μm prefilter before 0.2 μm filtration. *S. sonnei* GMMA are then purified by TFF (300 kDa cut-off, TMP 0.5 barg) as follows: a first diafiltration step against Tris buffer (50 mM Tris pH 7.5 with 2 mM MgCl_2_) followed by a recirculation step in Tris buffer containing Benzonase (10,000 U/L) and then a second diafiltration step against 10 mM Tris buffer with 0.7 M NaCl at pH 7.5 to reduce nucleic acids and soluble protein content. GMMA are finally exchanged in 10 mM Tris buffer pH 7.5 with 0.15 M NaCl to produce the final bulk.

### GMMA characterization

Total OAg content is quantified by HPAEC-PAD, total protein content by micro BCA and OAg/total protein ratio is calculated. Lipid A amount is quantified by HPLC-RP MS, while particle size is determined by dynamic light scattering or HPLC-SEC/MALS. The percentage of soluble proteins is determined by protein quantification in the supernatant after GMMA ultracentrifugation. The OAg is extracted after treatment with acetic acid and characterized for O-acetyl content by NMR or Hestrin colorimetric method. OAg molecular size distribution is measured by HPLC-SEC/semicarbazide, and Lipid A structure is confirmed by MALDI-TOF. All methods are previously described^41^.

### altSonflex1-2-3 formulation

The altSonflex1-2-3 vaccine is formulated to contain equal amount of OAg from each of the four GMMA components (i.e., 15 µg OAg from GMMA from each strain, *S. sonnei* NVGH2929, *S. flexneri* 1b NVGH2858, *S. flexneri* 2a NVGH2404 and *S. flexneri* 3a NVGH2766) (60 µg OAg in total) in a 0.5 mL dose. The formulation procedure was developed at a small scale and scaled up to 3 L to produce altSonflex1-2-3 GMP lots. In more detail, GMMA are diluted with saline to reach a final concentration of 600 µg OAg/mL (150 µg/mL of each OAg), and subsequent 0.22 µm filtered with Sartobran P150 filter. GMMA are then diluted with WFI under constant stirring at 90 rpm to reach a final concentration of 120 µg OAg/mL (30 µg/mL of each OAg) in the final formulated bulk. Alhydrogel is added to reach a final concentration of 0.7 mg Al^3+^/mL in the final formulated bulk, followed by continuous stirring at 90 rpm for 2 h. Then 100 mM sodium phosphate buffer pH 6.5 is added to reach 10 mM in the final formulated bulk, followed by continuous stirring at 90 rpm for 15 min. Finally, NaCl solution 1540 mM is added to reach 154 mM in the final formulated bulk, followed by continuous stirring at 180 rpm for 15 h ± 4 h.

### altSonflex1-2-3 characterization

*S. sonnei* OAg and total *S. flexneri* OAg were quantified by HPAEC-PAD analyses performed directly on Alhydrogel formulation as previously described^41^. Each single OAg component was instead quantified by FAcE^69^. Total protein content was estimated by micro BCA^31^. Micro BCA was also performed on Drug Product supernatant collected after 0.2 µm centrifugal filtration by nanosep of the vaccine sample together with HPLC-RP/MS to quantify unabsorbed protein and lipid A GMMA contents, respectively^41^. Light scattering was used for particle size estimation^70^. MAT was performed as previously described^66^.

### Immunogenicity studies

GSK is committed to the Replacement, Reduction and Refinement of animal studies (3Rs). Non-animal models and alternative technologies are part of our strategy and employed where possible. When animals are required, the application of robust study design principles and peer review minimizes animal use, reduces harm and improves benefit in studies.

Animal studies were performed in GSK Animal Facility (Siena, Italy) or in Charles River Laboratories (France) in compliance with the relevant guidelines (Italian D.Lgs. n. 26/14 and European directive 2010/63/UE) and the institutional policies of GSK. The animal protocol was approved by the Italian Ministry of Health (AEC project No. 1140/2020-PR, approval date 18/11/2020).

To assess the immunogenicity of altSonflex1-2-3, groups of 8 CD1 female mice 4-6 weeks old or 8 New Zealand white rabbits were immunized IP and IM respectively with different doses of the vaccine candidate in 200 μL (mice) or 500 μL (rabbits) total volume. All doses were prepared by dilutions with Alhydrogel in 10 mM sodium phosphate buffer pH 6.5 and 154 mM sodium chloride (0.7 mg Al^3+^/mL). In some experiments corresponding mono-component formulations were also tested. Animals were immunized twice at days 0 and 28, and blood was collected at different time points and the serum obtained. IgG antibodies elicited to *S. sonnei* LPS and *S. flexneri* 1b, 2a and 3a OAg were assessed by ELISA. ELISA plates were coated with LPS/OAg (LPS for *S. sonnei*, OAg for *S. flexneri*), blocked with PBS milk 5%, and incubated with the sera diluted 1:100, 1:4000 and 1:160,000 in PBS-Tween 0.05% 0.1% BSA (for mouse sera) or BS milk 5% (for rabbit sera). Bound antibodies were then detected using an enzyme-labeled secondary antibody (anti-mouse or anti-rabbit IgG-alkaline phosphatase) in PBS-Tween 0.05% 0.1% BSA. The presence of immunoreacting anti-*S. sonnei* LPS/*S. flexneri* 1b, 2a, 3a OAg IgG was detected by the addition of substrate solution, and the formation of yellow color was detected by absorbance at 405 nm subtracted by the absorbance at 490 nm. The samples were tested in comparison to calibrated mouse or rabbit anti-antigens specific reference standard sera. Results were expressed in ELISA units/mL determined relative to the reference serum. One ELISA unit equals the reciprocal of the dilution of the reference serum that yields an OD of 1 in the assay.

Individual serum samples were also tested against wild-type bacterial strains in SBA based on luminescent readout as previously described^71^. Results of the assay were expressed as the IC50, the reciprocal serum dilution that resulted in a 50% reduction of luminescence and thus corresponding to 50% growth inhibition of the bacteria present in the assay. Conditions used were optimized for each bacterial strain in terms of the percentage of Baby Rabbit Complement used and buffer of the assay as previously reported^39^. GraphPad Prism 7 software was used for curve fitting and IC50 determination. A titer equal to half of the first dilution of sera tested was assigned to titers below the minimum measurable signal.

### Toxicity of altSonflex1-2-3 vaccine in rabbits

To support the clinical administration of up to three immunizations of altSonflex1-2-3, a toxicology study was conducted with male and female New Zealand White rabbits in compliance with Good Laboratory Practice (GLP) standards (Charles River Laboratories, France). The vaccine was administered four times, 2 weeks apart by the IM clinical route and at the highest planned clinical dose of 60 µg total OAg in 0.5 mL. A control group received saline at the same administration schedule, route and injectable volume. A 4-week observation period after the last injection was observed. Rabbits were selected as the animal model based on preliminary research studies demonstrating the capability of the vaccine candidate to produce an immune response. All animals were observed during the course of the study for morbidity/mortality, clinical signs, injection sites evaluation (Draize), ophthalmology, body weights and body weight gains and food consumption. Clinical pathology, immunogenicity evaluation, necropsies macroscopic and microscopic observations (WHO tissue list), and organ weights were also performed. Body temperatures were also recorded at baseline (three times), right before each injection and three times after each injection day.

### Statistical analysis

Mann–Whitney two-tailed test was used to compare the immune response elicited by two different formulations at the same time point. Spearman rank test was used to verify dose response. Statistical analysis was performed using GraphPad Prism 7.

### Reporting summary

Further information on research design is available in the Nature Research Reporting Summary linked to this article.

### Supplementary information

Supplementary Material

Reporting Summary

## Data Availability

All data associated with this study are present in the paper or the Supplementary Materials. Requests for resources, data and reagents should be directed to the corresponding author. Material Transfer Agreements would be required for any sample requests.
