# Supplementary material for: A next-generation GMMA-based vaccine candidate to fight shigellosis
Source: NPJ Vaccines. 2023 Sep 5;8:130. doi: 10.1038/s41541-023-00725-8 (PMC10480147; doi:10.1038/s41541-023-00725-8)
Supplement: Supplementary file 1 — Supplementary Material [file 41541_2023_725_MOESM1_ESM.pdf]

Supplementary Figures

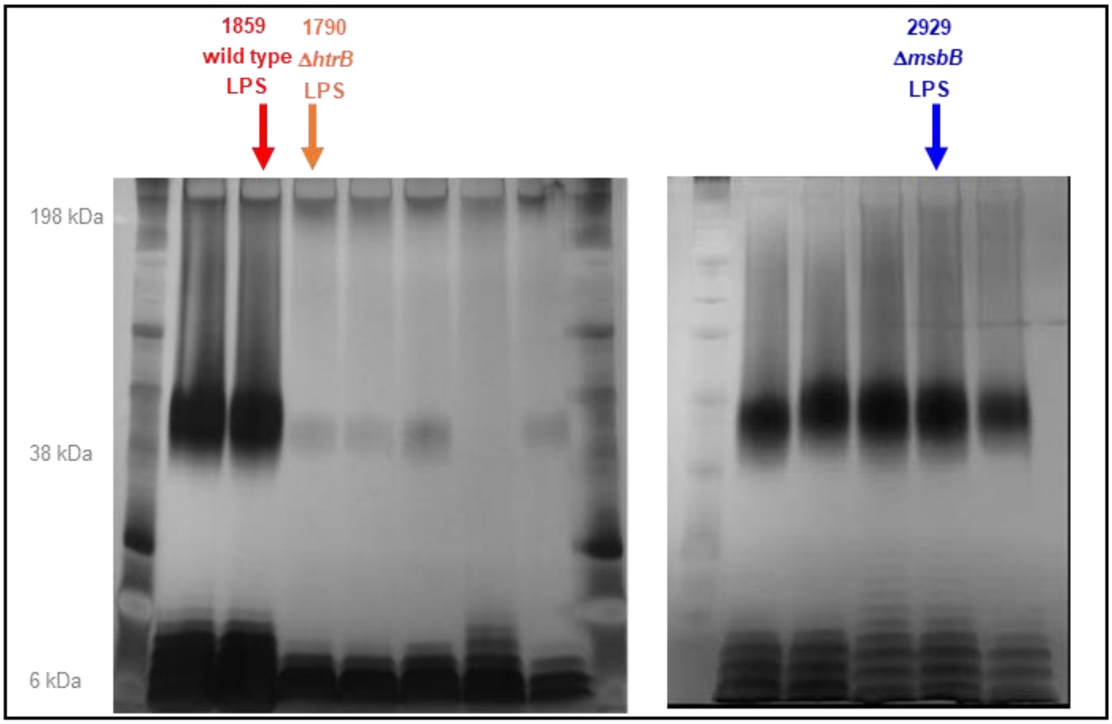

Supplementary Figure 1. Uncropped SDS-PAGE silver stained of LPS extracted from GMMA producing bacteria normalized at same OD; Gels derive from the same experiments and have been processed in parallel. With arrows are indicated the lanes showed in Figure 1b: red arrow indicates “wildtype 1859 LPS”, orange arrow indicates “ 1790 Δ*htrB* LPS”, blue arrow indicates “ 2929 Δ*msbB* LPS”.

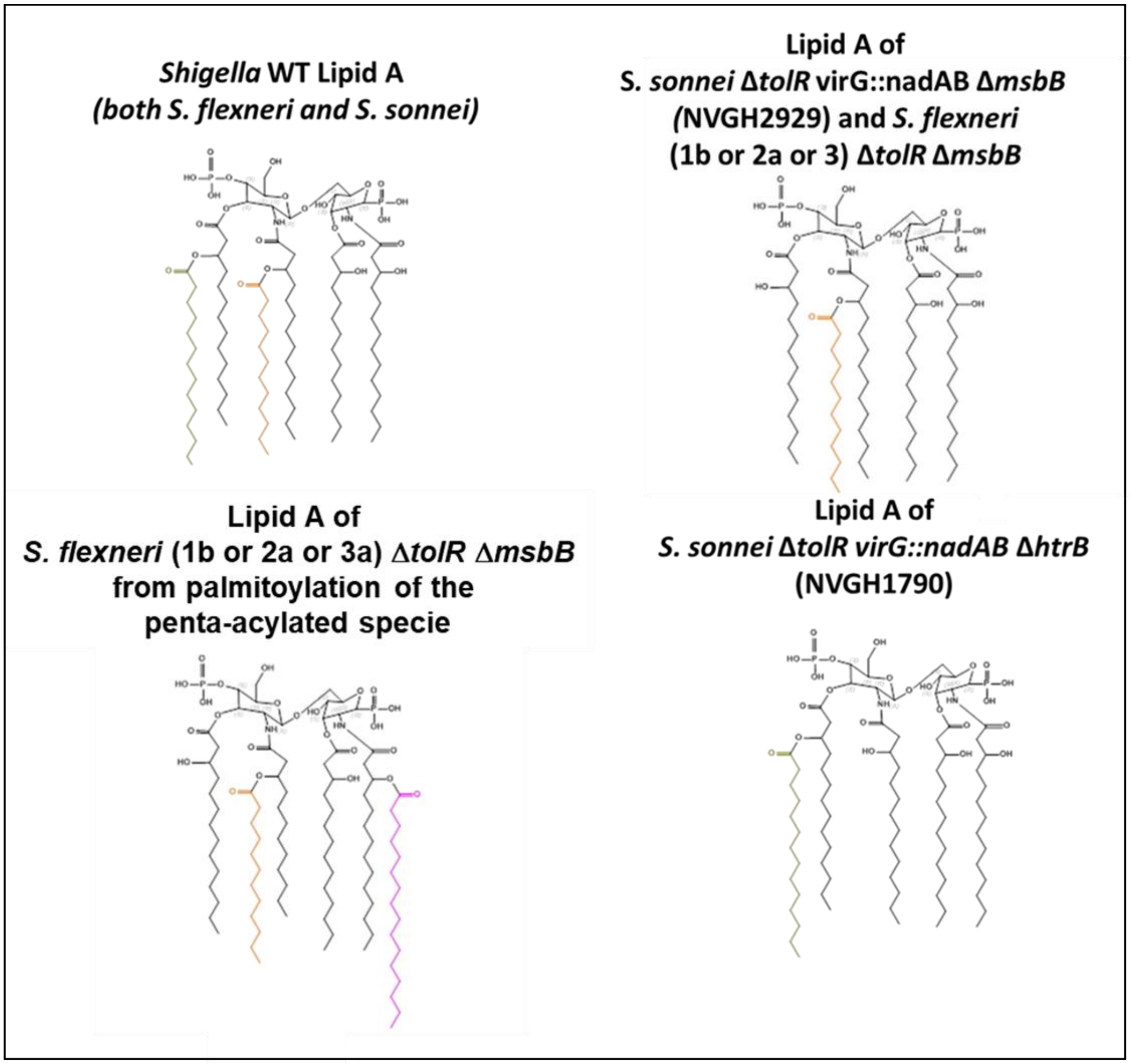

Supplementary Figure 2. Lipid A structures present in wild type *Shigella* strains and in the corresponding mutated strains for GMMA production.

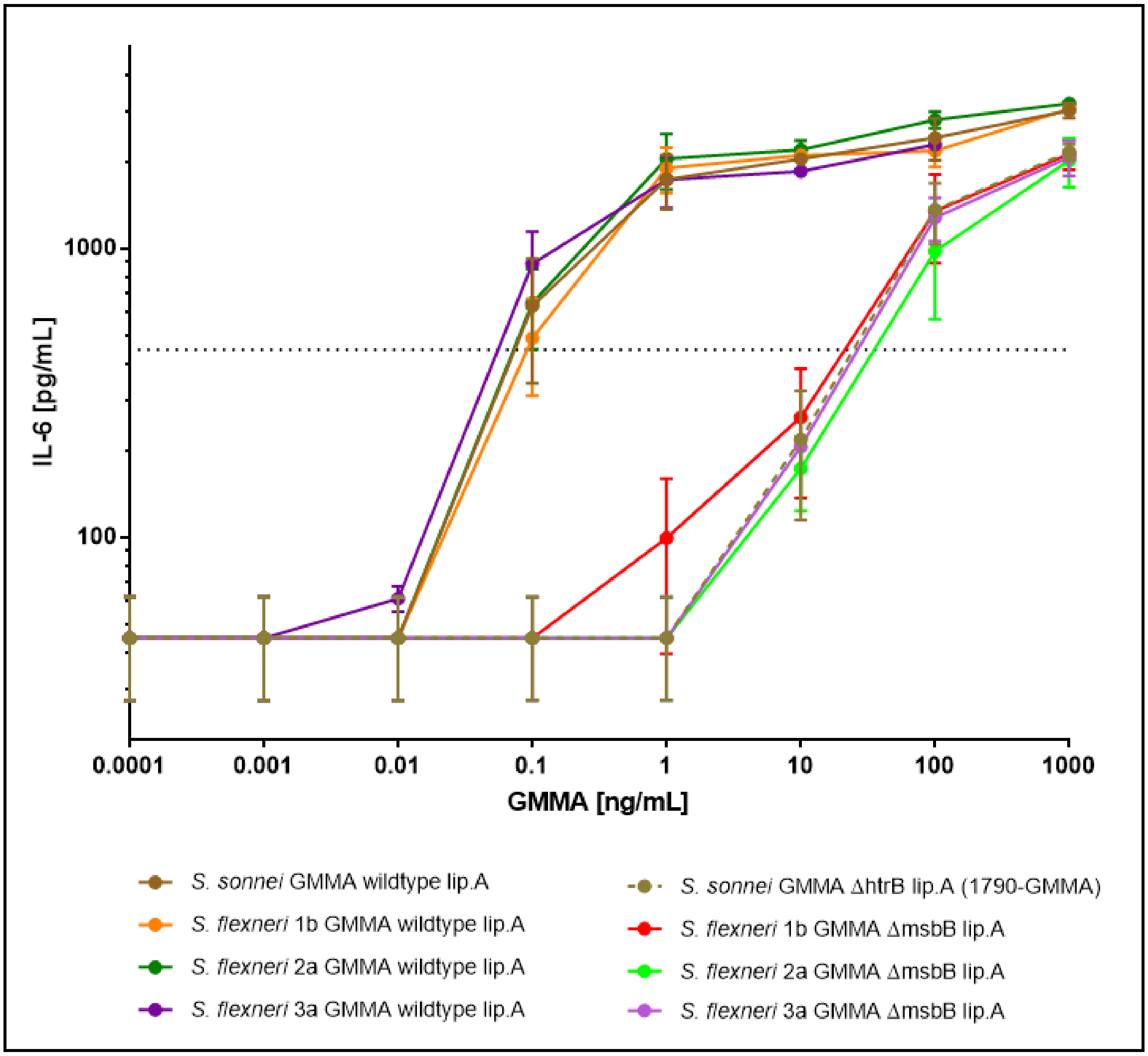

Supplementary Figure 3. MAT assay: IL-6 release from human PBMC exposed to *S. flexneri* GMMA with modified lipid A in comparison to corresponding GMMA with wild type lipid A and *S. sonnei* 1790-GMMA.

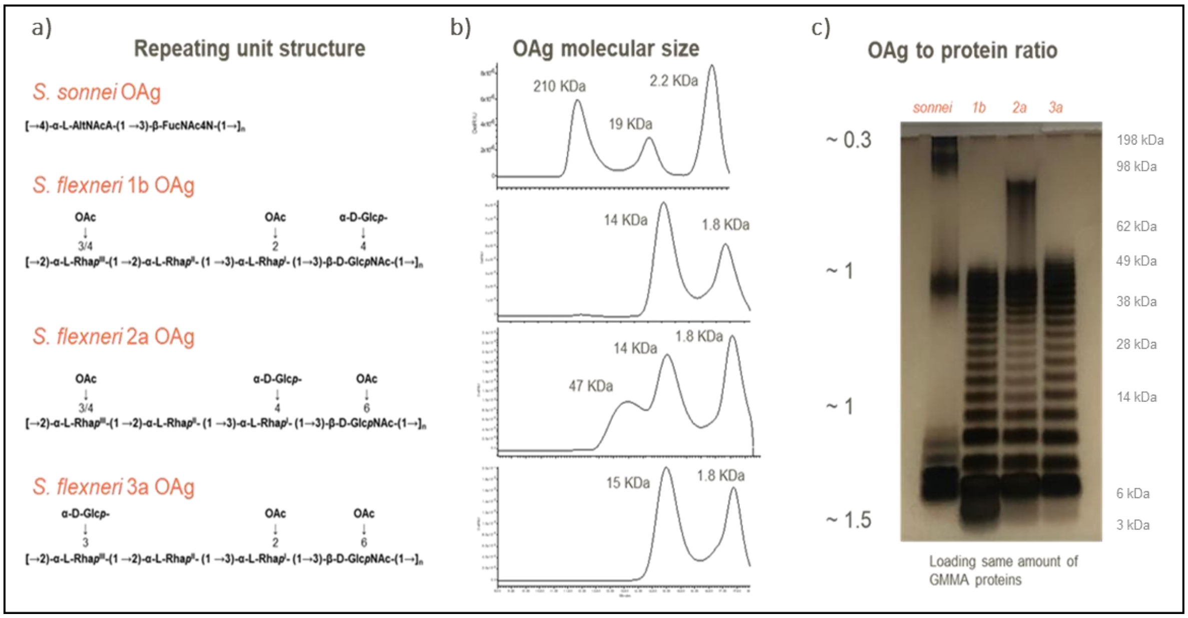

Supplementary Figure 4. OAg repeating unit structure of the 4 different *Shigella* GMMA present in altSonflex1-2-3 (a); molecular size of each GMMA OAg as estimated by HPLC-SEC dRI profiles (b); SDS-PAGE silver staining of LPS extracted from same protein amount of the different GMMA (c). Gels derive from the same experiments and have been processed in parallel.

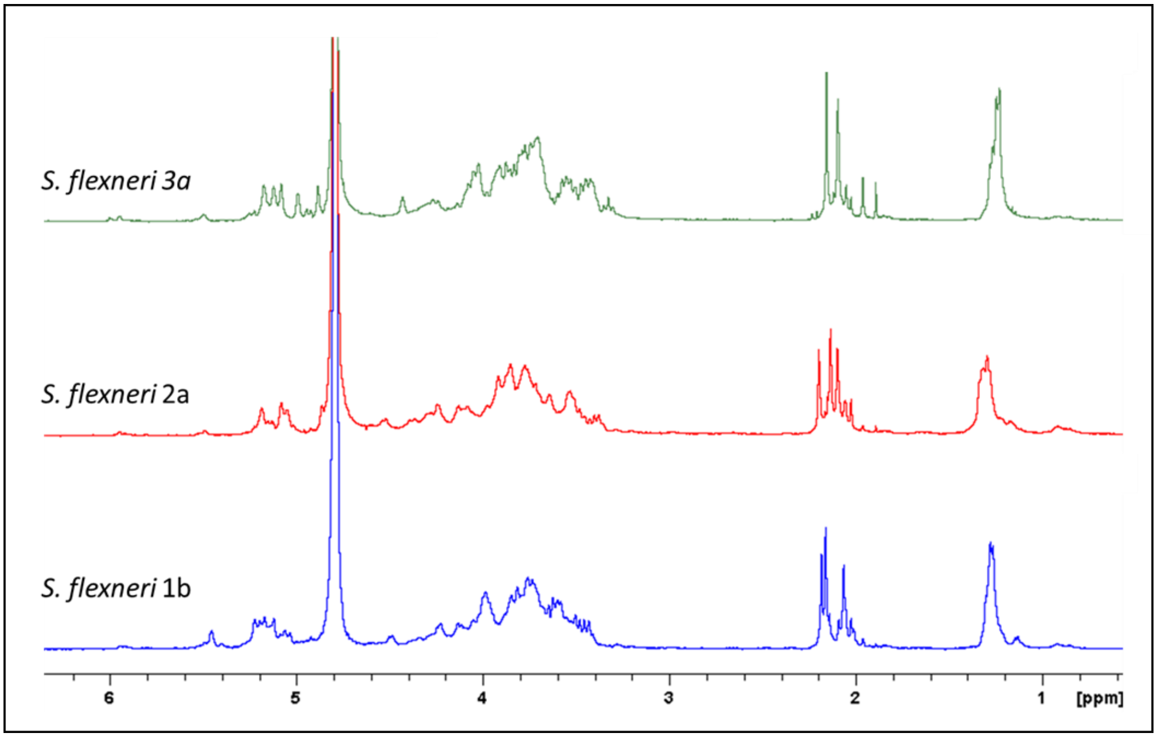

Supplementary Figure 5. ^{1}H NMR of OAg extracted from *S. flexneri* 1b, 2a and 3a GMMA.

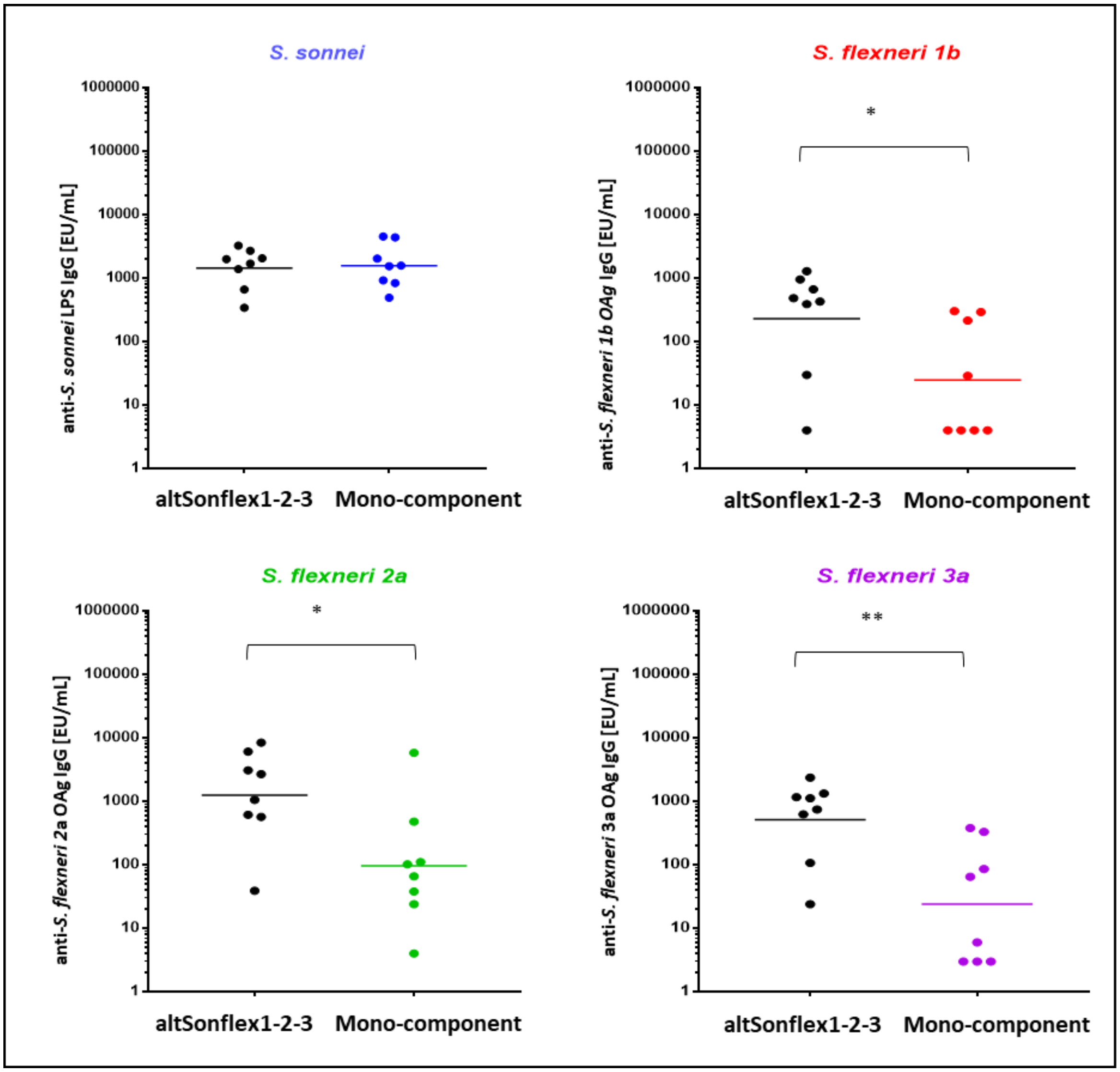

Supplementary Figure 6. Anti-OAg IgG responses elicited in mice 27 days after first injection by altSonflex1-2-3 compared to corresponding mono-component formulations at 9.4 ng each OAg dose. CD1 mice were immunized IP at day 0 and 28. Geometric mean (bar) is reported for all groups together with individual values (dots).

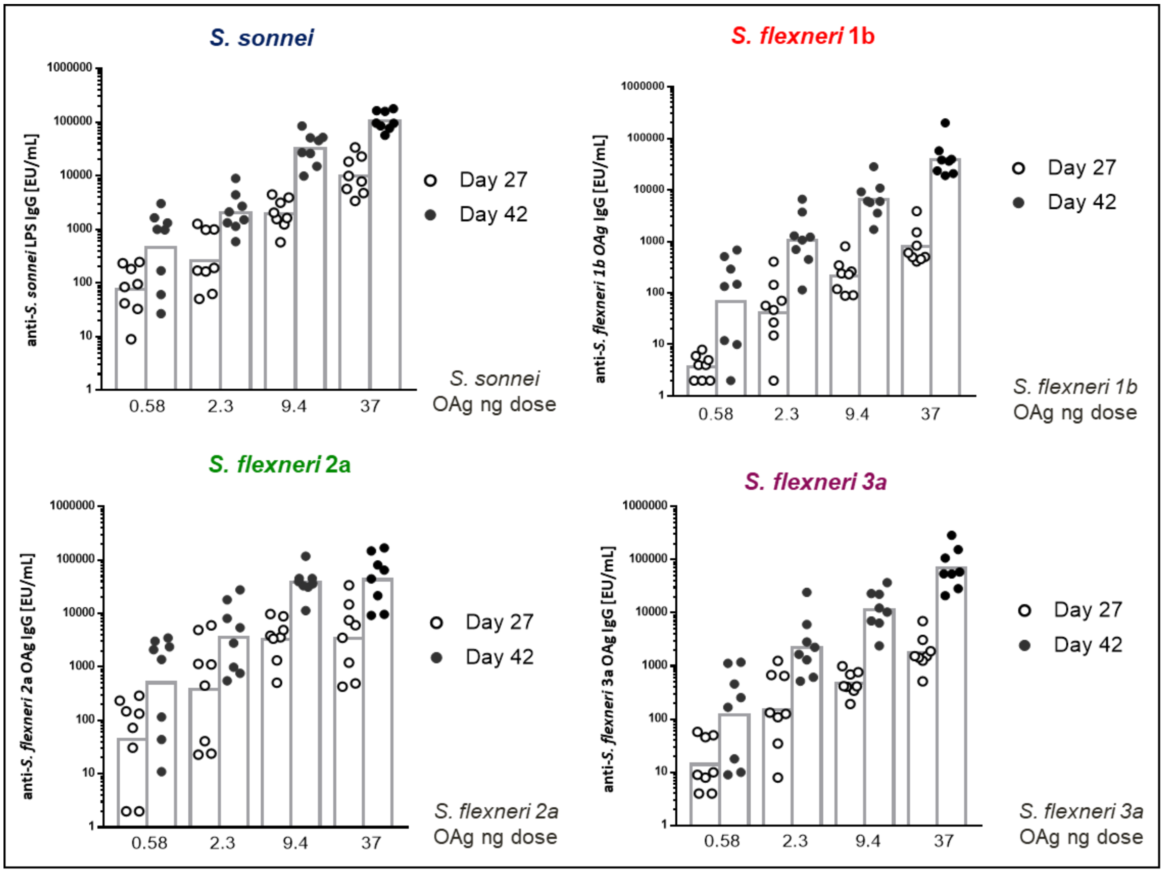

Supplementary Figure 7. Dose-dependent anti-OAg IgG responses elicited in mice by altSonflex1-2-3. CD1 mice were immunized IP at days 0 and 28 and sera analyzed at days 27 and 42. Geometric mean (bar) is reported for all groups together with individual values (dots).

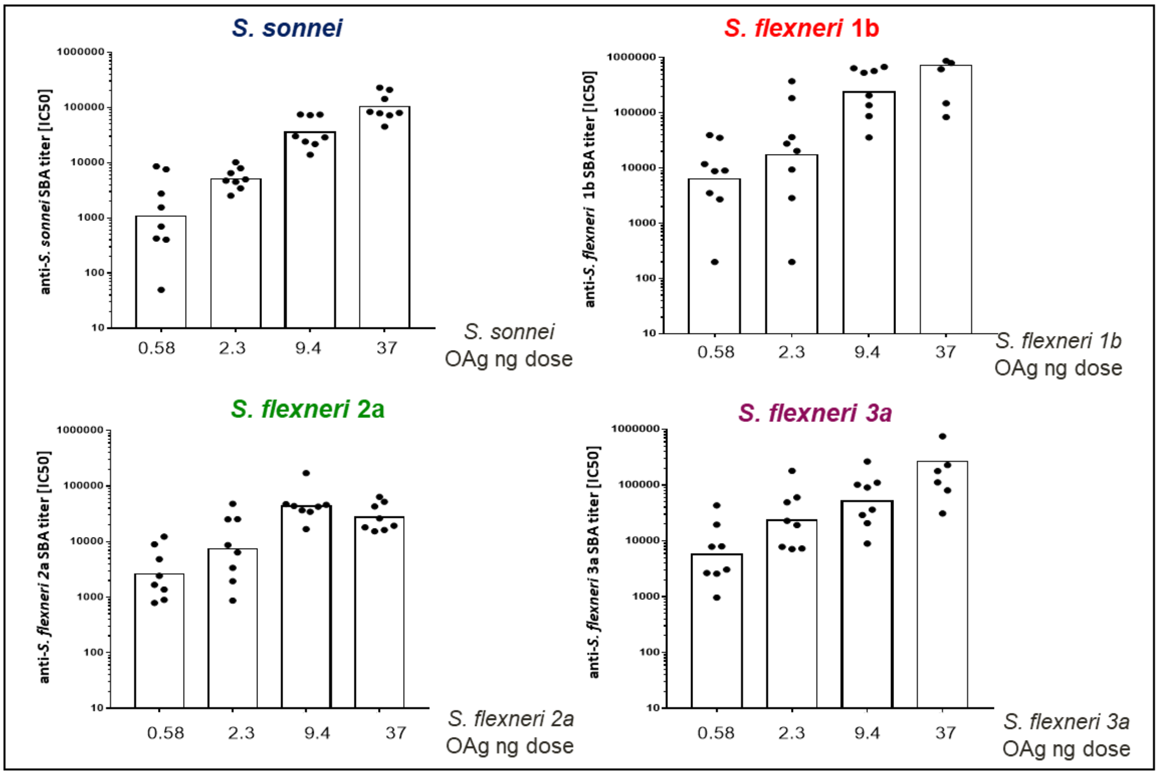

Supplementray Figure 8. Dose-dependent SBA titers elicited in mice by altSonflex1-2-3. CD1 mice were injected IP twice at days 0 and 28, and sera were collected at day 42 to assess functionality by SBA. Geometric mean (bar) is reported for all groups together with individual values (dots).

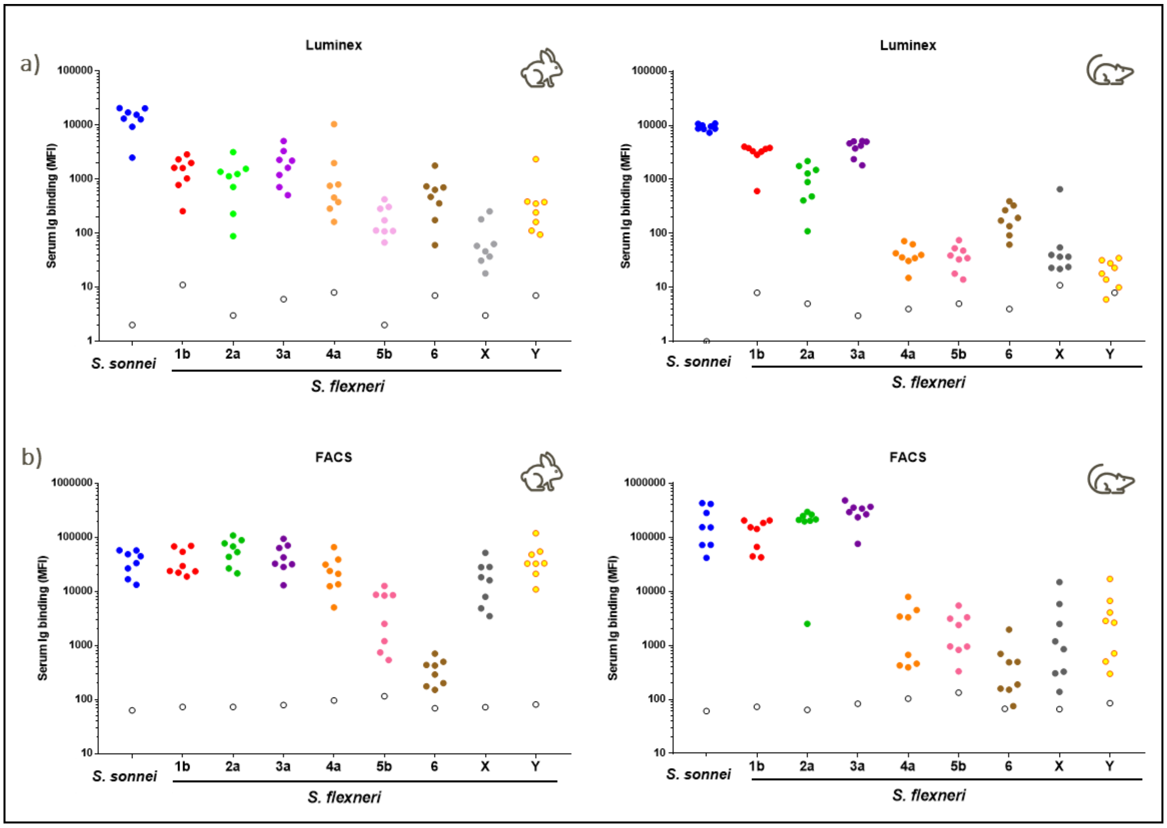

Supplementary Figure 9. Sera from mice and rabbits were tested at dilution 1:500 by Luminex **(a)** and FACS **(b)** against epidemiologically relevant *Shigella* O-Ag/wild-type strains. Sera at day 42 from mice immunized IP at days 0 and 28 with 0.06 μg each OAg and from rabbits immunized IM at days 0 and 28 with 1.5 μg each OAg were tested. White dots refer to Mean Fluorescent Intensity (MFI) of pre-immune sera.

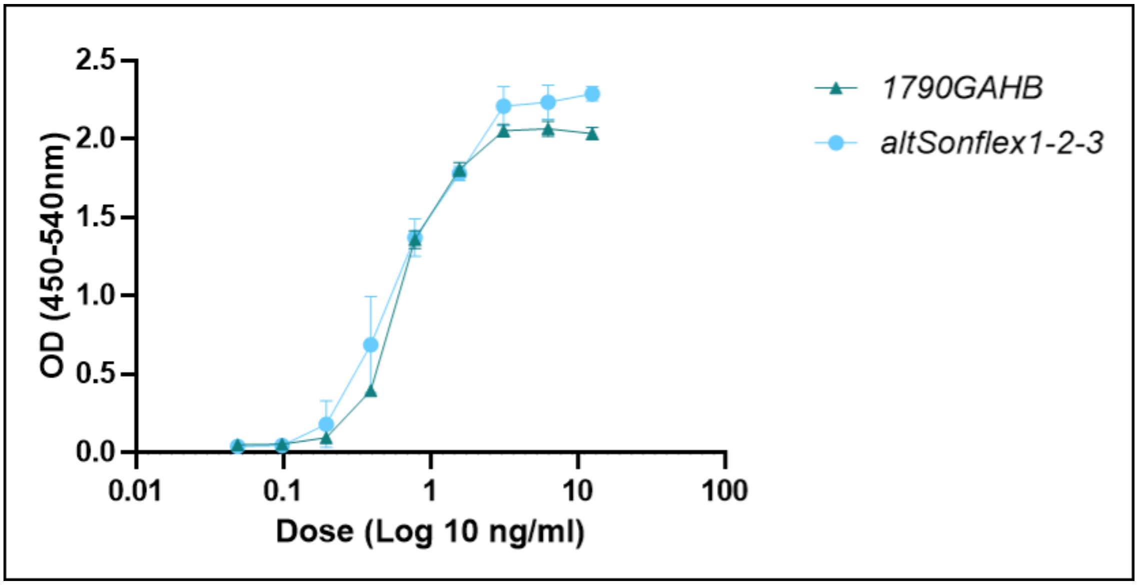

Supplementary Figure 10. altSonflex1-2-3 induces similar proinflammatory cytokine release from human PBMC as 1790GAHB by MAT. Vaccine candidates were compared by starting dilutions from corresponding full human doses of 60 µg total OAg for altSonflex1-2-3 and 6 µg OAg for 1790GAHB. PBMCs from four donors were used; one representative donor is shown.

Supplemetary Tables

Supplementary Table 1. Characterization of *S. sonnei* GMMA with wild-type (1859-GMMA) and modified lipid A (1790-GMMA and 2929-GMMA). In depth characterization of OAg populations displayed on GMMA surface is reported.

**GMMA**	**1859**	**1790**	**2929**
OAg/protein weight ratio[1]	0.21	0.03	0.29
G4C/HMW OAg (200-240 kDa)[2]	8%	8.9%	48.0%
MMW OAg (19-20 kDa)[2]	74%	76.9%	32.7%
LMW OAg (2.2 kDa)[2]	18%	14.0%	19.1%
LipidA/protein ratio (nmol/mg)	287	197	144
Particle size (Z average diameter nm)	103.4<br>0.209	118.9<br>0.219	160.4<br>0.160

[1]Protein amount estimated by micro BCA for 1859- and 2929-GMMA and by Lowry for 1790-GMMA.[2] % of each population at different size on total OAg+G4C extracted (OAg and Group 4 Capsule share same repeating unit structure).

Supplementary Table 2. *S. sonnei*, *S. flexneri* 1b, 2a and 3a GMMA characterization.

Test	Method	*S. sonnei* GMMA	*S. flexneri* 1b GMMA	*S. flexneri* 2a GMMA	*S. flexneri* 3a GMMA
OAg/protein weight ratio	HPAEC-PAD and micro BCA	0.29	1.2	1.1	1.2
Soluble protein/total protein %	micro BCA	5.7	1.4	2.0	1.2
Lipid A/OAg (nmol/µg)	HPLC-RP MS/ HPAEC-PAD	0.5	0.39	0.22	0.37
Lipid A/total protein (nmol/µg)	HPLC-RP MS/micro BCA	0.14	0.46	0.25	0.43
OAg molecular size distribution (KDa)	HPLC-SEC	234<br>19.2<br>2.2	13.8<br>1.7	47.1<br>14.2<br>1.8	15.5<br>1.8
Particle Size<br>Z-average (diameter in nm)<br>PDI	Dynamic light scattering	160.4<br>0.160	108.9<br>0.18	109.7<br>0.14	82.5<br>0.17

Supplementary Table 3. 2929-GMMA *S. sonnei* batch produced at 30 L scale.

**Test**	**Method**	***S. sonnei*** **Development batch**	***S. sonnei*** **Reference Std. batch**	***S. sonnei*** **Development batch**	***S. sonnei*** **GMP batch**
Total OAg/total protein (w/w ratio)	HPAEC-PAD/ micro BCA	0.32	0.29	0.26	0.23
Soluble protein/total protein (%)	micro BCA	0.8	5.7	5.5	2.0
Lipid A/OAg (nmol/mg)	HPLC-RP MS/ HPAEC-PAD	0.6	0.5	0.6	0.87
Lipid A/total protein (nmol/mg)	HPLC-RP MS/ micro BCA	0.2	0.2	0.2	0.2
OAg molecular size distribution (kDa)	HPLC-SEC	G4C/HMW 247.3<br>MMW 18.7<br>LMW 2.2	G4C/HMW 234<br>MMW 19.2<br>LMW 2.2	G4C/HMW 201<br>MMW 19.5<br>LMW 2.2	G4C/HMW 209.6<br>MMW 19.6<br>LMW 2.3
Particle Size<br>Z-average diameter (nm);<br>PDI	Dynamic light scattering	162.8<br>0.206	160.4<br>0.160	154.1<br>0.210	141.8<br>0.198

Supplementary Table 4. *S. flexneri* batches produced at 30 L scale.

Test	Method	***S. flexneri* 1b Reference Std. batch**	***S. flexneri* 1b Development batch**	***S. flexneri* 1b GMP batch**	***S. flexneri* 2a Reference Std batch**	***S. flexneri* 2a Development batch**	***S. flexneri* 2a GMP batch**	***S. flexneri* 3a Reference Std batch**	***S. flexneri* 3a Development batch**	***S. flexneri* 3a Development batch**	***S. flexneri 3a* GMP batch**
Total OAg/total protein (w/w ratio)	HPAEC-PAD micro BCA	1.2	1.2	0.9	1.1	1.1	1	1.2	1.2	1.6	1.6
Soluble protein/total protein (%)	micro BCA	1.4	1.5	6	2.0	2.9	7	1.2	0.8	0.6	<3
OAg O-Acetyl content (Molar ratio OAc/Rha)	Hestrin-Dische	50.1	60.6%	51[1]	48.7%	54.8%	64	33.6%	41.3%	31.0%	38[1]
Lipid A/OAg (nmol/µg)	HPLC-RP MS/HPAEC-PAD	0.39	0.39	0.5	0.22	0.28	0.4	0.37	0.35	0.29	0.4
Lipid A/total protein (nmol/µg)	HPLC-RP MS/micro BCA	0.46	0.48	0.46	0.25	0.29	0.33	0.43	0.44	0.46	0.66
OAg molecular size distribution (KDa)	HPLC-SEC	MMW 13.8<br>LMW 1.7	MMW 13.7<br>LMW 1.8	MMW 13.0<br>LMW 3.0	HMW 47.1<br>MMW 14.2<br>LMW 1.8	HMW 52.0<br>MMW 13.7<br>LMW 1.7	HMW 59.0<br>MMW 14.0<br>LMW 2.0	MMW 15.5<br>LMW 1.8	MMW 15.3<br>LMW 1.8	MMW 15.1<br>LMW 1.8	MMW 15.0<br>LMW 3.0
Particle Size Z-average diameter (nm); PDI	Dynamic light scattering	108.9<br>0.18	113.4<br>0.17	110<br>0.19	109.7<br>0.14	112.5<br>0.14	117.0<br>0.16	82.5<br>0.17	81.5<br>0.20	87.7<br>0.16	88<br>0.21

[1]values collected at 12 months real time stability

Supplementary Table 5. Characterization of different altSonflex1-2-3 lots produced at different scale.

Test	Method	Toxicology batch (1L scale)	altSonflex 1-2-3 GMP batch (1L scale)	altSonflex 1-2-3 GMP batch (3L scale)
OAg quantity (*S. sonnei* and total *S. flexneri*)	HPAEC-PAD	nd	31 µg/mL (*S. sonnei* OAg) 77 µg/mL (*S. flexneri* OAg)	34 µg/mL (*S. sonnei* OAg) 81 µg/mL (*S. flexneri* OAg)
OAg quantity of each GMMA component	FAcE	*S. sonnei* OAg: 29 µg OAg/mL *S. flexneri* 1b: 38 µg OAg/mL *S. flexneri* 2a: 36 µg OAg/mL *S. flexneri* 3a: 31 µg OAg/mL	*S. sonnei* OAg: 31 µg OAg/mL *S. flexneri* 1b: 35 µg OAg/mL *S. flexneri* 2a: 39 µg OAg/mL *S. flexneri* 3a: 31 µg OAg/mL	*S. sonnei* OAg: 28 µg OAg/mL *S. flexneri* 1b: 32µg OAg/mL *S. flexneri* 2a: 31[1] µg OAg/mL *S. flexneri* 3a: 29 µg OAg/mL
pH	Potentiometry	6.6	6.5	6.6
Osmolality	Osmometry	302 mOsm/kg	306 mOsm/kg	310 mOsm/kg
GMMA (lipid A) not adsorbed on Alhydrogel[1]	HPLC-RP/MS	2.9 nmol/mL	2.4 nmol/mL	1.2 nmol/mL
Total Protein not adsorbed to Alhydrogel	micro BCA	7.0 µg/mL	9.4 µg/mL	9.4 µg/mL
Particle size D[4,3] and D(90) [1]	Light Scattering	D[4,3]: 4.4 µm D(90): 7.2 µm	D[4,3] 4.7 µm D(90) 7.7 µm	D[4,3] 6.9 µm D(90) 9.1 µm

[1]value collected at 6 months real time stability time point.

Supplementary Table 6. Primers used in the generation of *S. flexneri* 1b.

1: virG-1	GTCACAGGTAACATGACTCTGGAG
2: virG-2	CCATGTGTGAATACTACCTTCACCC
3: ospD3-1	GTTTTGCCTCATTCAAGATATCACC
4: ospD3-2	TGACGATGGTTTGTCAGGATTGC
5: msbB.F	CGCCAAAGTTCCGTGATCCCATT
6: msbB.R	CTCTTCGATGATCTCCAGCCCCTT
